# Report of the Meeting of the Medical Association of the Southwest

**Published:** 1918-11

**Authors:** 


### The Medical Association of the Southwest Holds Annual Convention in Dallas, Texas.

The last meeting of the Medical Association of the Southwest was held in Dallas, Texas, October 15-17, inclusive.

An unusually interesting program would have made it one of the best meetings ever held by the society, had not the influenza epidemic, at this time sweeping with such force all over the territory, interfered with prearranged plans, forbidding the attendance of many members and physicians appearing upon the program. Added to this was the fact that a decided number of the members were seeing active service in war work. While the attendance was small, great enthusiasm was displayed by the members and guests present in the activities of the Association.

Instead of having three sections carrying on meetings simultaneously, it was decided to alternate the program with papers from the different sections each day, particularly as much time was to be given over to a discussion of war topics, as they concerned physicians at this time. The various section officers presided during the three days.

The membership of the Medical Association of the Southwest is 967, with approximately 200 physicians serving with the army.

The Oriental Hotel was the official headquarters, and the meetings were held in the Junior Ball Room of the Adolphus Hotel.

At the opening ceremonies, Dr. M. M. Smith of Dallas, chairman of the Committee on Arrangements, called the meeting to order and introduced the various speakers.

Patriotic singing, led by Rev. William M. Anderson, opened the exercises, which was followed by prayer.

Addresses of welcome were made by Dr. Minnie L. Maffett, vice-president of the Dallas County Medical Society; Mayor Joe E. Lawther, and Dr. H. Leslie Moore, president District Medical Society.

The general plan of clinics for the meeting was dispensed with, on account of influenza situation, which kept all the local physicians busy, but Major John O. McReynolds, M. C., U. S. A., invited all present at the morning session to visit the special laboratories at St. Paul's Sanitarium, where special care is given the examination of applicants or entrants to the aviation branch of the service. Clinics were conducted there every afternoon.

One of the special features of the program was the address of Judge Quentin D. Corley, official representative of the American Red Cross, on Vocational Rehabilitation. Judge Corley illustrated his lecture and gave a very practical demonstration of how successfully this work may be carried on by means of his own specially prepared apparatus. (Judge Corley had the misfortune of losing his left forearm and the entire right arm, with part of that shoulder blade.) By means of the mechanical attachments which he uses, he can do practically what any other person can do—such as dressing and feeding himself, driving his own auto, taking care of his garden, attending to his office duties, writing a clear, legible hand, etc., etc. Judge Corley has tendered the use of his patented devices to the Government in its scheme of rehabilitation for crippled soldiers.

Lieutenant Colonel Harry E. Mock, M. C., U. S. A., while unable personally to attend the convention, sent in a most interesting paper on "Reconstruction work in U. S. Hospitals," which was illustrated with moving pictures showing the work being carried out at the Walter Reed Hospital in Washington, D. C.

Mr. Thomas J. Taylor, U. S. Revenue Agent from San Antonio, spoke of his experiences with the enforcement of the Federal Anti-narcotic law and its present gross violation by unscrupulous physicians, druggists, etc., throughout this section of the country. He begged the cooperation of ethical physicians in helping him to see to it that this wise law was better observed.

Food Administrator Harry E. Barnard, for the State of Indiana, ex-president of that State Board of Health, spoke most eloquently on "A Word Concerning the Problem Confronting the U. S. Food Administration."

"A Method of Controlling Preventable Diseases in Rural Districts," was ably discussed by Dr. A. Caswell Ellis, of the University of Texas. He gave a recital of the work done in connection with certain health surveys made in different counties, where the expense was jointly borne by the Extension Department of the Texas State University, the International Health Commission (Rockefeller Fund)' and the various counties in which the work was carried on.

The last day's meeting was devoted to a symposium or round table discussion of Spanish influenza and its complications. This discussion was supposed to have been opened by Health Commissioner John W. Duke, of the State of Oklahoma, but a wire from him announced that the epidemic in the territory under his individual jurisdiction forbade his leaving to attend the meeting.

Major J. O. McReynolds of Dallas, presented this subject for discussion, relating his personal experience with influenza at Camp Dick. A lively discussion followed, which was participated in by Dr. Wm. A. Davis, Secretary of the Texas State Board of Health, and most of the other physicians present. Among the points of special interest brought out, were; first, that both the very old and the very young, appear to resist the disease better than do those of early adult life. Dr. J. C. Loggins, physician in charge of the Confederate Home at Austin, reported that though the youngest inmate of that institution was 71 years of age, only two cases had as yet made their appearance there and they soon recovered. It was further brought out that while a number of children had taken influenza, complications were not frequent and even when they appeared they were not usually fatal. The appearance of these types of the disease, that were marked by extereme nervous symptoms, that of the gastro-intestinal type, and broncho-pneumonia, was noted. The method of transmission was judged to be atmospheric, rather than by contact and for that reason it was not considered a strictly quarantinable disease.

On Wednesday evening, Oct. 16, a lecture with lantern slides, by Dr. C. A. R. Campbell of San Antonio, Texas, was given on "The Eradication of Malaria by the Cultivation of Bats—the Mosquitoes' Natural Enemies." This was a recital of Dr. Campbell's 16 years of study and experimental work along this line, and it seems that he has clearly proven the success of his methods, as evidenced by the municipal bat roosts now maintained by the City of San Antonio. While the initial cost for building these is considerable, yet they soon get to be self-sustaining, as the sale of the bat guano will bring in something like $200 annually from each roost.

Dr. Campbell offers to the scientific world a wonderful advance along constructive lines, looking to the prevention of malarial fever and the prompt and effectual destruction of the recognized conveyors of this disease and his work is in time destined to receive the commendation and praise of health officials all over the country.

The concluding feature of the three-days' session was the four-reel Government war film—"Fit to Fight," sent to Dallas under the direction of the Medical Section of the Council of National Defense. This is the best educational work looking to the prevention of venereal diseases that has appeared, and should be shown at all military camps and as soon as time will permit, to all the growing youths of our land.

All papers appearing on the program, whose authors were not able to attend the convention, were read by title and voted to be published in the regular proceedings.

The meetings while small in point of attendance, were rich in scientific value.

Oklahoma City, Okla., was selected as the convention city for 1919.

The following officers were elected to serve during the coming year:

President—Dr. M. M. Smith, Dallas, Texas.

Vice Presidents—Dr. L. von Treba, Chetopa, Kans.; Dr. O. B. Hall, Warrensburg, Mo.; Dr. F. W. Jelks, Hot Springs, Ark.; Dr. F. E. Camp, Oklahoma City, Okla.

Secretary Treasurer—Dr. F. H. Clark, El Reno, Okla.

Chairman, Committee on Arrangements—Dr. Everett S. Lain, Oklahoma City, Okla.

